# Under careful construction: combining findings, arguments, and values into robust health care coverage decisions

**DOI:** 10.1186/s12913-022-07781-1

**Published:** 2022-06-07

**Authors:** T.H. Kleinhout-Vliek, A.A. De Bont, A. Boer

**Affiliations:** 1 Erasmus School of Health Policy & Management, Erasmus University, P.O. Box 1738, 3000 DR Rotterdam, the Netherlands; 2 Copernicus Institute of Sustainable Development, Utrecht University, Utrecht, the Netherlands

**Keywords:** Health Care Decision-making, Health Care Coverage, Expertise, Patient and Public Involvement and Engagement, Robustness

## Abstract

**Background:**

Health care coverage decisions deal with health care technology provision or reimbursement at a national level. The coverage decision report, i.e., the publicly available document giving reasons for the decision, may contain various elements: quantitative calculations like cost and clinical effectiveness analyses and formalised and non-formalised qualitative considerations. We know little about the process of combining these heterogeneous elements into robust decisions.

**Methods:**

This study describes a model for combining different elements in coverage decisions. We build on two qualitative cases of coverage appraisals at the Dutch National Health Care Institute, for which we analysed observations at committee meetings (*n* = 2, with field notes taken) and the corresponding audio files (*n* = 3), interviews with appraisal committee members (*n* = 10 in seven interviews) and with Institute employees (*n* = 5 in three interviews), and relevant documents (*n* = 4).

**Results:**

We conceptualise decisions as *combinations of elements*, specifically (quantitative) findings and (qualitative) arguments and values. Our model contains three steps: 1) identifying elements; 2) designing the combinations of elements, which entails articulating links, broadening the scope of designed combinations, and black-boxing links; and 3) testing these combinations and choosing one as the final decision.

**Conclusions:**

Based on the proposed model, we suggest actively identifying a wider variety of elements and stepping up in terms of engaging patients and the public, including facilitating appeals. Future research could explore how different actors perceive the robustness of decisions and how this relates to their perceived legitimacy.

## Introduction

Health care coverage decisions specify whether or not a specific health care technology is to be provided or reimbursed at a national level. In many cases, a document with reasons for these decisions is available to the public. Various elements may be present in such a document. The (generally quantitative) evidence collated and generated through Health Technology Assessment (HTA) is but a part of this: many formalised criteria also contain qualitative considerations [1–4]. In addition, some authors are advocating “evidence-informed evaluation of [previously] identified [stakeholder] values” [5, 6], while others work on integrating and accounting for value plurality [7]. Moreover, a growing body of literature establishes the impact of additional, non-formalised or ‘contextual’ factors on these decisions, often noting inconsistencies in the use of formalised criteria [8–12] (10.1016/j.healthpol.2012.09.012). In short, many heterogeneous elements may be present in the decision making and the public report. These elements have typically been brought together through a deliberative process.

Since the turn of the century, scholars have closely examined deliberative processes on health care coverage [13–15], showcasing the interactional work required during deliberation [16, 17]. In essence, this interactional work comprises clustering, i.e., establishing coherence between these heterogeneous elements [18–20]. These heterogeneous elements would (ideally) be contributed by a diverse set of actors and combined into what Science and Technology Studies (STS) scholars have called a ‘robust’ decision [21–27].

Many authors working inductively on these deliberations have shown how they involve combining rationales. These authors generally draw conclusions at a relatively broad level, not infrequently classifying deliberations and their outcomes as ‘pragmatic’ [26, 28–31]. Russell devised and employed a rhetorical policy analysis method to study deliberations, concluding that decisions are ‘practical productions’ interwoven with ‘big D’ policy discourse [32]. We owe much to her work on how different quantitative and qualitative elements, criteria and case-specific arguments, are routinely combined into decisions that are both ‘rational’ and ‘human’ [28]. Russell did not, however, concern herself with distinguishing generalisable *steps* in clustering the variety of elements into a decision.

In this paper, we construct a model for clustering elements during deliberations on health care coverage, drawing from literature on decision making that yields outcomes that are ‘robust’, that is, able to withstand pressure in society. Such pressure may take the form of media attention or even public controversy, which has previously resulted in the reversal of decisions, generally through a direct appeal to the Minister of Health or other public authority responsible [33, 34]. Relatively robust decisions would not generate such pressure or would be able to withstand it.

Our model will conceptualise decisions, i.e., the publicly available reasons provided for a decision, as *combinations of elements*. The element types we distinguish are findings, arguments, and values. We describe three steps to achieving robust decisions: 1) identifying elements, 2) designing combinations of elements, and 3) testing these different combinations. We will illustrate this model with qualitative comparative case research data on two Dutch health care coverage decisions, namely on maternity care and paracetamol and vitamins. In the Netherlands, like in many other countries, the HTA body (the Dutch National Health Care Institute) is responsible for combining such elements into a decision containing publicly available reasons. Notably, in the Dutch system, this is an *advised* decision, as the Minister of Health takes the final decision. S/he generally follows the advised decision but sometimes deviates from it [35]. The cases both concern a decision that needed to be revisited (for different reasons) and thus are likely to be relatively carefully made [12]. This analysis results in several recommendations for policy and research.

## Three-step model: robust decisions as combinations of elements

STS scholarship considers controversies to be fruitful sites for exploring technology's role in society [36, 37]. Scholars have described how polities have dealt with controversies as diverse as nuclear power plants, radioactive waste storage, Bovine Spongiform Encephalopathy, HIV/AIDS, Genetically Modified Organisms, nanotechnology, and coverage decisions [24, 26, 38]. These studies describe the work to uphold or defuse a controversy, highlighting the insufficiency of traditional, ‘certified’ expertise, always making the reader sensible to the many ways controversies come to be and in which closure was perhaps achieved [36].

Rip [21, 22, 25] advocates focusing on the production of ‘robustness’. Generally, STS scholars define robustness as ‘surviving’ public pressure or incorporating ‘non-certified’ expertise [23, 39]. Rip embarks upon his operationalisation of robust decisions through ‘informal technology assessment’, in essence, a public litmus test for decisions. Robust outcomes, he poses, can withstand “the pressures to which they will inevitably be exposed” [21, 27].

According to Rip, these decisions may contain:

“arguments, evidence, social alignments, interests, and cultural values, many of them interrelated and therefore lending support to the dominant view. The difference between an only fashionable and a robust view is a matter of degree, and perhaps also a matter of actual effort that actors are prepared to exert.” [22]

Robust decisions thus contain heterogeneous elements, and actors need to exert substantial effort to identify these elements. Thus, the first step of our model is: *identifying potentially relevant elements*. Elsewhere, Rip speaks of “findings, arguments, perceptions, interests, and dominant values” [25]. We focus on three elements generally present in health care coverage decisions: a reduction of the variety of elements present. These elements are 1) findings of experts in the shape of Health Technology Assessments and the like; 2) arguments such as what concerns good care; and 3) values such as justice, equality, and solidarity [2, 20, 30, 40, 41].

The second step we derive from literature is: *designing linked combinations of elements*. As Rip continues,

“[Both fashionable and robust views] are available in the cultural repertoire, but with increasing robustness, the linkages between elements of the view and with their context increase in number and in articulation (and sometimes also in scope).” [22]

Rip highlights the availability of different possible combinations of elements, which differ in robustness. Some are on the “fashionable” end of the spectrum, while others are more robust. To achieve a robust view, whoever is arguing needs to *link* elements into clusters of factors [18, 19]. We conceptualise these linked sets of elements as ‘combinations’. Setting up robust combinations of elements involves matching up these elements [22]. In a similar vein, Callon, Lascoumes and Barthe advocate “the *design* and testing of (…) [multiple] solutions that integrate a plurality of points of view, demands, and expectations” [24].

Rip distinguishes two different linking activities, namely articulation and consolidation. By articulation, he understands that the speaker actively joins previously unlinked elements and that this may also result in an increase in number. In some cases, this may also involve increasing the scope of the decision. Such an increase in scope may take the form of comparing or connecting in other elements not usually considered relevant for this type of decision (cf. ‘contextual’ or case-specific factors mentioned above). Consolidation, for Rip, is the next step in robustness, linking several elements so firmly together that the combination becomes a ‘black box’ and, as such, may be routinely used as a standing combination of elements which is difficult to call into question [22]. Black-boxing links may happen in the decision making moment, or decision makers may use previously-formulated black boxes. Coverage decisions usually contain several such combinations of elements, for instance, the ‘incremental cost-effectiveness ratio’ [42]. In sum, designing the combination of elements takes three forms in our model: active articulation of links, increasing the links in number and sometimes in scope, and black-boxing the links.

After identifying the elements and designing multiple combinations, our model's third step is to *test the combinations of elements*. Callon et al., first put forward testing different combinations as a series of negotiations and compromises between all present [24]. They argue that these should be part and parcel of the decision-making process instead of remaining informal and outside the formal procedures. Testing different combinations is to be actively encouraged in a ‘safe’ space as increasing numbers of participants, with a variety of perspectives, acquire a stake and a voice. Callon et al. envision this to happen in *hybrid forums*, where technical experts and other stakeholders design and test several combinations of elements together [24]. Such learning is consistent with Rip's earlier work [22]; Nowotny [23] conceptualises robustness as resulting specifically from such repeated testing.

We will show how the decision trajectories of two specific cases, namely the Dutch health care coverage decisions on maternity care and paracetamol and vitamins, illustrate this three-step model as they link multiple, heterogeneous elements into relatively robust decision combinations.

## Comparative case methodology

**Fig. 1:**
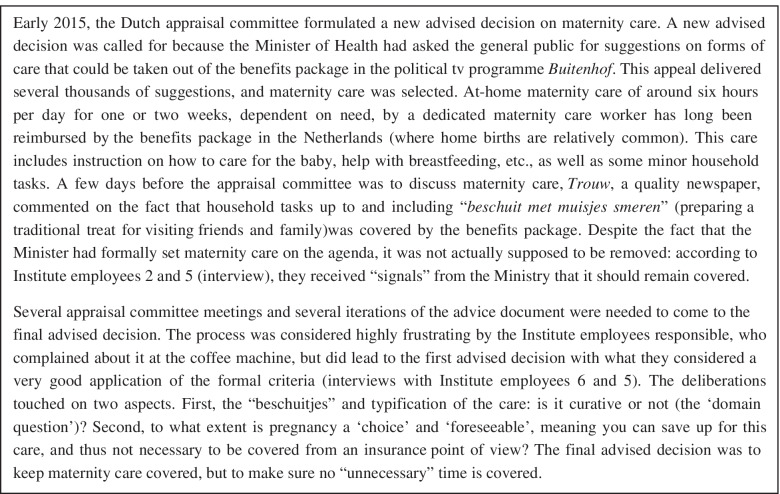
Maternity care case description

**Fig. 2:**
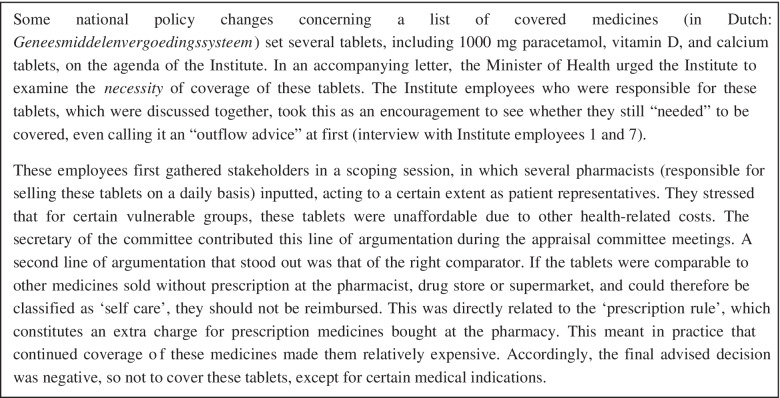
Paracetamol-vitamins case description

This paper builds on research at the Dutch Health Care Institute and employs a case approach [43, 44]. Case analysis is well-placed to provide insights into health care coverage decisions as it gives an in-depth take on processes that entail valuation [20, 45, 46]. We opted for two highly contrasted cases [47] regarding the decision, the number of patients affected, the type of technology, and the price: maternity care and paracetamol and vitamins (for more information, see Figs. 1 and 2) [48]. The Dutch media discussed both cases briefly [49, 50]. Both cases were revisited decisions and were already in the basic benefits basket, but this status was now questioned [12]. Given this history, these are cases where decision makers are likely to have constructed the decision especially carefully.

The data consisted of observations at the appraisal committee meetings (*n* = 2, both for paracetamol and vitamins, with field notes taken) and analysis of the corresponding audio files (*n* = 3, with one for the maternity care case and the other two of the observed meetings), interviews with committee members (*n* = 10 in seven interviews) and with Institute employees (*n* = 5 in three interviews). The interviews were semi-structured and ‘field formal’ [51, 52]. We also analysed four documents pertaining to the two decisions. The secretary of the appraisal committee granted access to the meetings and the (audio) files. Previous observations at appraisal committee meetings (*n* = 2) enriched the data analysis (see Tables 1 and 2).

**Table 1:** Overview of documents and audio files analysed per case

Case study	Documents and audio files
Maternity care	discussion document 1.1 appraisal report 1.2 patient contribution (part of discussion document) 1.3 audio file 1
Paracetamol and vitamins	discussion document 2.1 appraisal report 2.2 audio file 2

**Table 2:** Overview of interviews and observations analysed

Interviews and observations date	Description
January 2015	Appraisal committee maternity care *audio file only*
March 2015	Institute employee 6
March 2015	Committee member 5
August 2015	Committee member 3
September 2015	Committee member 2
November 2015	Appraisal committee paracetamol and vitamins *observations and audio file*
October 2016	Committee member 5
October 2016	Institute employees 1 & 7
October 2016	Institute employees 2 & 5
October 2016	Appraisal committee paracetamol and vitamins *observations and audio file*
February 2017	Committee members 1, 4, 5 & 6
February 2017	Committee member 1
October 2017	Committee member 6

## Dutch health care coverage decisions

In the Netherlands, all citizens are insured through private insurance, which covers at least the bare minimum set by the government: the basic benefits basket. The Dutch Health Technology Assessment (HTA) agency, the National Health Care Institute (in Dutch: *Zorginstituut Nederland*, in the rest of this paper: ‘the Institute’), is responsible for formulating ‘advised decisions’ to the Minister of Health regarding the contents of this benefits basket. The Institute utilises four formalised criteria to come to this advice. These are: 1) effectiveness and 2) cost-effectiveness of the health care technology, 3) feasibility of coverage (including total budget impact), and 4) necessity, which rests in part on the individual severity of illness and individual affordability [53]. These well-established criteria [54] are brought together in the final advised decision sent to the Minister, who decides whether to follow the advice.

The process of coming to these advised decisions comprises several steps, relatively common in its set-up [55, 56]. Generally, it starts with a scoping session inviting stakeholders to submit initial comments on the health care technology under consideration. An assessment phase follows, wherein the scientific evidence, which includes information on the effectiveness, cost-effectiveness, budget impact, severity of illness, and affordability, prepared by Institute employees, is examined by the assessment committee (in Dutch: *Wetenschappelijke Adviesraad*). The appraisal committee (in Dutch:* Adviescommissie Pakket*) subsequently contextualises the outcomes of this examination. In this meeting, which specifically aims to ‘bring in’ broader societal values, the scientific evidence is first presented by an Institute employee, followed by input from patient (representative)s and other stakeholders. Subsequently, the appraisal committee, which comprises eight to ten external experts from fields such as pharmacoeconomics and health care ethics, commences their deliberations. At the end of these deliberations, which allow each member to speak and respond, the committee formulates its final advice. This advised decision is then summarised, discussed and approved by the Institute’s Board of Directors, and forwarded to the Minister [57, 58].

## The three-step model in practice

Our conceptual model comprises three steps to be taken in deliberation resulting in robust decisions, which we conceptualise as combinations of heterogeneous elements. The first step is the identification of different elements (findings, arguments, and values). Second, combinations of elements are designed by linking elements, broadening the combination's scope, and ‘black-boxing’ links. In the third and final step, decision makers confront or ‘test’ the combinations for robustness and choose one combination as the final health care coverage decision.

### Step 1: Identifying elements

The first step in coming to a robust decision is to identify the various elements that may make up the combination. Identifying ‘all’ elements is not possible; instead, decision makers’ efforts in this area are rewarded with many different elements and many different types of elements. One way of obtaining elements is by inviting experts with experience and other stakeholders, such as patients or other members of the public, into the deliberation. In the Dutch appraisal committee, deliberations do start with contributions from Institute employee(s) and, sometimes, patient (representative)s. The appraisal actively invites these contributions, and treats them as valuable, especially in structuring the deliberations to achieve agreement about the advice to be offered to the Minister [31, 59].

These contributions contain the three types of elements we identified: findings, arguments, and values, visible in the dataset on maternity care (see Fig. 1) and paracetamol and vitamins (see Fig. 2). *Findings* included the individual severity of illness and the financial cost of the paracetamol and vitamins for the patient if the benefits were excluded. These were contributed by the Institute employee. *Arguments* included one to the effect that maternity care workers have an essential signalling function to other health care professionals when there are problems or high-risk situations with the mother and baby, necessitating new arrangements if the benefit were to be excluded. For paracetamol and vitamins, arguments included that the pharmacists considered it likely that patients would opt for heavier medicines still covered by the benefits package once the paracetamol and vitamins were no longer covered. In both cases, *values* mentioned by a committee member included solidarity with vulnerable groups such as chronically ill, elderly patients, or new mothers who might not be able to afford maternity care (audio files 1 and 2).

This overview and some of the data below demonstrate that the source of arguments for the committee’s consideration were sometimes patients and newspaper articles. Such dynamics raise questions of the identity and distinctiveness of expertise and its role in policy-making – not for nothing has this been a long-standing debate [21, 39, 60–64]. We follow Callon et al. [24] and Moreira [26, 27] in not assessing quality differences between types of contribution and recognising that a larger number of participants is likely to yield more elements. These may clash with one another; some will argue in favour of and others against coverage of this particular health care technology [65]. However, such clashing may not be problematic as they may become part of different combinations (see step 2 below).

### Step 2: Designing combinations of elements

The second step is to design the combinations of elements [24]. This step is divided into three distinct activities: articulating links between elements, broadening the combination's scope, and black-boxing links [22].

*Articulating links* is the primary method for connecting elements into decisions [18, 19]. The decision report always contains a variety of formalised criteria and case-specific considerations [12, 31]. Links are constantly made in deliberations. We are specifically interested in links made between different types of elements. The paracetamol-vitamins case gives an example:

“People who take these medicines often have more costs due to comorbidity and/or cannot afford them because of a low average income coupled to lower socioeconomic status” (Discussion document 2.2).

As this served as argument for coverage, this is an explicit linking of equity (people with lower socioeconomic status or other costs should also be able to take these medicines) with the finding of the severity of illness (specifically: comorbidity) and the argument personal responsibility (they should thus not be personally responsible). In the maternity care case, a strongly linked combination was already available before the deliberations started but it was explicated during the deliberations because maternity care arrived on the decision agenda of the Institute in an unusual way (see Fig. 2). In *Buitenhof*, a well-known political tv programme, the Minister of Health had asked stakeholders and members of the public what forms of care would not, in their opinion, need to be covered by the benefits basket. Maternity care was selected out of 3921 suggestions received by the Minister. A few newspapers picked this up. The line of argument, this linked combination, was formulated by a committee member during the committee deliberations as follows:

“There are signals that solidarity no longer goes without saying. To put it bluntly: “I do not think I shall need maternity care for the rest of my life, so I’m not going to pay for it”. [Some commotion from the rest of the committee, committee member 8 is invited to continue]. “Things could be a bit more nuanced. If you look at that article in *Trouw* [Dutch newspaper] of this week, following the draft advice that was released, you'll see that maternity care is associated straightaway with prepping *beschuitjes*.” [Laughter] “And if at that point someone would say, “Wait a minute, er, should I pay for that?”, I would have some sympathy with that.” (Committee member 8, audio file 1)

This comment has a direct impact on the final decision document, where the following line was added:

“[In terms of] necessary maternity care for which we show solidarity, we envision care with a medical purpose and not the image of the maternity care worker who serves *beschuit met muisjes*.” (Discussion document 1.2)

This combination of quotes shows that *different* combinations play a role in the deliberations [24], and the decision-making process and the final decision benefit from linking these elements and contrasting these with other combinations in situ. It also highlights the expertise brought to bear in these processes.

*Broadening the combination's scope* appears rarely in the published literature, where decisions hinge on explicated reasons and rarely concern other health care areas [22]. However, the coverage decisions studied contain recommendations, and we pose this may fall in this category [31]. In the paracetamol and vitamins case, the primary rationale was that reimbursing these medicines would make them more expensive due to the fact that pharmacists charge extra for formally prescribed medicines: the ‘prescription rule’ (see Fig. 2). One of the appraisal committee members broadened the scope of the coverage decision, formulating it as if directly giving the Minister of Health advice on the prescription rule:

“You may make many more [medicines] available outside the pharmacy. Given the situation, this is our answer: if it has to be bought at the pharmacy, it has to be reimbursed. But we advise you to think carefully about the prescription rule because that creates a completely unequal ratio between those cheap medicines that are and those that are not available on prescription.” (Committee member 6, audio file 2)

Giving the Minister advice on the prescription rule has little to do with determining the basic benefits basket: it falls outside the appraisal committee's remit. However, this type of recommendation provides an strengthening element of a combination. The scope of the decision is broadened by going beyond the coverage decision. Specifically, the direction the combination is broadened in through such recommendations remains at the committee’s discretion. Sometimes they formulate advice to the Minister, sometimes to other stakeholders. In this way, the committee not only specifies what a good basic benefits basket is but also what good *care* entails, thereby actively broadening the decision's scope and the committee’s remit.

*Black-boxing links* is the final and the most robust aspect of designing combinations. The example provided by Rip concerns the link between smoking and cancer [22]. Black-boxing links even more uncharted territory than broadening the scope of the combination when it comes to health care coverage decisions. In fact, given the strong history of elaborate reasonings that explicate many elements and links between elements [66, 67], the idea of actively ‘obscuring’ links and making them hard to call into question may seem counter-intuitive. However, cost-effectiveness in and of itself could be seen as black box, linking many separate elements such as quality-adjusted life years, costs per treatment, and effectiveness, into a widespread coverage criterion [54]. In this sense, the committee’s deliberations (almost) always employ a black box. Black-boxing links is also visible to some extent in the appraisal committee’s work we have studied. The paracetamol and vitamins case featured the rule of thumb ‘cheaper than €100 per year means no coverage’; the individual responsibility for the ‘bottom’ (cheaper end) of the benefits basket is noted, informally, to apply to any medicine cheaper than €100 per year. This rule of thumb is a black box in the sense that it is *not done* to question it. We observed how one committee member did question this link tentatively, only for another to answer:

“We don’t want a discussion on what price is affordable (lit: how much money can come for own account)” (Committee member 5, audio file 1)

Black-boxing links thus happens (but is naturally not explicated), and these links are difficult to prise open.

### Step 3: Testing combinations

The third and final step is to test these different decision combinations. The fact that different combinations may exist, and that one needs to be chosen, has previously been described for a decision for a costly treatment, which gained a positive coverage status because one set of clustered argumentations together weighed more heavily than another set [18, 19]. Callon et al. describe this process as a series of negotiations and compromises in a hybrid forum setting, which harnesses learning as part of the decision process [24, 26]. Interaction between technical experts and other stakeholders is vital in this learning process. In the paracetamol and vitamins case, pharmacists, patient organisations, medical specialists, and a pharmaceutical company argued in favour of coverage. As committee member 5 summarised on behalf of these groups:

“It concerns largely vulnerable groups, chronically ill elderly patients, people who are regularly on the receiving end of budget cuts, have little money, often deal with the accumulation of costs (…) this is the most necessary care, and that should be covered per definition, as it is, erm, cost-effective care with which you can prevent [additional] costs that might occur later on, for example when these people do not take these medicines and get fractures, [which is] miserable for those people [but] also means additional costs.” (Committee member 5, observations/audio file 2)

This potent alternative combination of taking care of vulnerable groups, arguing in favour of coverage, required an equally strong combination arguing against coverage. Committee member 4 achieves this by making *more* links to the former combination and then dismissing this combination by noting it as another’s responsibility:

“Through the argument of not being able to afford [the tablets] it seems as if (…) there is some sort of poverty boundary where people through the calcium tablets will suddenly end up on the wrong side. But in those cases, (…) there is probably already more going on, with those people, already the government, all kinds of related measures, rent subsidies, benefits, are happening. And it won’t [mean] those benefits agencies will give extra benefits because of this tablet, but there is a whole host of expenses, gas, light, and oh yes, the costs of this medicine, so there’s much more to it. I think it is almost, how should I say it, almost a self-centred idea that we or the [collective health] insurance were going to make the difference between poverty and no poverty. So, I would argue to leave the poverty and the not being able to afford [things] to agencies that deal with these things, [because] it will not be influenced by that one-and-a-half calcium tablet (…). You can talk about those 100 euros, but it is always, low cost – just put it aside.” (Committee member 4, audio file 2).

First, this is a notable remark as it narrows the committee’s responsibility, counter to broadening it (see above). Second, this combination was quickly considered to be decisive, with little discussion; the committee responded primarily by noting that this problem should not indeed be solved through health insurance (Committee member 6, audio file 2) and the fact that “the whole system is inefficient” (Committee member 4, audio file 2). Different combinations were also available in the repertoire in the maternity care case, as the alternative element combination placed it on the agenda, as described above. Having summarised this alternative line of argumentation based on newspaper *Trouw,* committee member 8 continues:

“[But] I think it's important to indicate something like: “Yes, but wait a minute, maternity care is about other matters, er, breastfeeding, detecting risky situations, etc., etc., for which it is completely just to be calling for solidarity”.” (Committee member 8, audio file 1)

This comment, which dismissed the element combination present in *Trouw* by decisively linking several essential elements together (breastfeeding, risk, solidarity), also had little subsequent discussion. It shows how different decision combinations are tested against each other in health care coverage decisions’ deliberative phase before one is chosen.

## Conclusion

Much inductive scholarship on health care coverage notes that decision-making processes feature many different considerations and may be classified as ‘pragmatic’. Most, however, do not necessarily seek structure in these processes. We have derived a model for making such decisions from Science and Technology Studies literature on robustness. By conceptualising decisions as combinations of heterogeneous elements (facts, arguments, values), we distinguish different ‘actions’ that decision-makers may take while deliberating: identifying elements, linking them together into combinations, and testing these combinations.

This model leads to three concrete recommendations: two for decision makers and one for research. First, we recommend that decision makers try to identify potential elements from a wider variety of sources. This work may include considering real-world data sources like social media [68] but as a source of additional decision elements rather than as input to be quantified or standardised into formalised criteria. This may also take the form of ‘horizon scanning’ (a term usually reserved for scanning for costly medicines about to enter the market) for potentially controversial decisions.

Second, decision makers should work towards approximating a hybrid forum-like setting for their decisions, enabling many more stakeholders to contribute. Achieving such a setting means opening the decision process (further) to specific personal interests, making some uncomfortable, who fear these interests may hijack deliberations [30]. We follow Rip and Callon et al. in considering personal interests un-extractable and even constructive to the decision-making process [22, 24]. Another objection to opening up the decision-making process is that the public engagement achieved may be no more than a legitimation exercise [61]. However, in our data, we saw that the establishment and testing of combinations did happen in the deliberative setting. It was not ‘for show’: the committee had not already decided beforehand. This shows that these meetings may indeed be a “forum for debating social desirability of innovations not generally deemed to be highly controversial” [14]. Such a hybrid forum-like setting should include an institutionalised appeals procedure for two reasons. The first is that consensus in such a forum gives “no guarantee that interests and concerns [have been] considered in the decision-making process” [64]. There are power differences inherent to these decision-making processes, and an appeals procedure gives an additional opportunity to confront a decision previously made by relatively powerful actors. Second, other combinations, other potentially robust decisions, are available for every decision made (see also the first recommendation). This warrants a securely institutionalised appeals procedure for re-examining decisions, especially as these may become outdated.

We thus broadly align with recent scholarship on evidence-informed deliberative processes [5, 6]. We would, however, pose that the ‘organic’ nature of decision-making processes as described above precludes the use of *checklists* of potentially relevant criteria. Such checklists leave little room for emotions and affect [69] and may obscure the power of rhetorics [32].

Third, further research should investigate the relationship between combination strength and how decisions’ robustness is perceived. Many STS scholars place the testing of the decision’s robustness outside the decision-making setting: in society, which indeed happens in health care coverage [33–35]. We consider learning more about decisions’ robustness as perceived by decision makers and other involved actors and its relation to decisions’ perceived legitimacy of vital importance.

## Data Availability

Under the research protocol as submitted to the Medical Ethics Committee at at Erasmus Medical Centre Rotterdam, the Netherlands, interview audio files and transcripts are kept confidential, and interviewees are anonymised in the manuscript. The datasets analysed during the current study are available from the corresponding author on reasonable request.

## Abbreviations

HTA: Health Technology Assessment
STS: Science and Technology Studies
